# Severe edema and blood blisters of the lower extremities caused by sitagliptin, a dipeptidyl peptidase‐4 inhibitor

**DOI:** 10.1002/jgf2.502

**Published:** 2021-10-14

**Authors:** Hiroki Maita, Tadashi Kobayashi, Takashi Akimoto, Hiroshi Osawa, Hiroyuki Kato

**Affiliations:** ^1^ Development of Community Healthcare Hirosaki University Graduate School of Medicine Aomori Japan; ^2^ Department of General Medicine Hirosaki University School of Medicine & Hospital Aomori Japan; ^3^ General Medicine Hirosaki University Graduate School of Medicine Aomori Japan

**Keywords:** blister, dipeptidyl peptidase‐4 inhibitor, edema, sitagliptin

## Abstract

Severe edema and blood blisters can occur as adverse events associated with sitagliptin. A history of dipeptidyl peptidase‐4 inhibitors should be considered when examining patients with edema and blood blisters of uncertain cause.

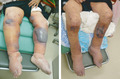

1

A 79‐year‐old woman admitted to a nursing home with hypertension, diabetes mellitus, osteoporosis, and constipation was referred to our hospital for severe edema and blood blisters of the lower extremities (Figure 1A). The patient was diagnosed with diabetes mellitus 4 years ago and had been prescribed sitagliptin for 3 years. She had well‐controlled blood pressure and blood glucose levels, and she weighed 54 kg before she began to experience symptoms. Approximately 3 years ago, she noticed slowly progressive edema in both lower extremities, and a detailed investigation was performed at a primary care hospital. Noncontrast computed tomography (CT) revealed a tumor in the left adrenal gland. Endocrinological evaluation revealed no hormonal abnormalities. She was diagnosed with a nonfunctional adrenal tumor. No other blood tests or lower limb venous ultrasound showed any obvious abnormality. A few months ago, her edema worsened, and bloody blisters appeared, which were not relieved by diuretics. She was referred to our hospital to investigate the cause of her symptoms. During the patient's first visit to our hospital, she was taking losartan, sitagliptin, spironolactone, lubiprostone, vitamin B complex, and potassium gluconate. Dipeptidyl peptidase‐4 inhibitor (DPP‐4i)‐induced bullous pemphigoid (BP) and edema were suspected, and sitagliptin was discontinued. Other drugs were administered, and no changes were noted. A week after the sitagliptin discontinuation, the edema significantly improved. No new blisters were observed, and her weight decreased from 62.3 to 55.5 kg (Figure 1B). At a follow‐up, her anti‐BP180 antibodies were negative, and BP was ruled out. The patient was diagnosed with DPP‐4i‐induced edema and blisters. Forty‐four days after the initial visit, her weight further decreased to 49.0 kg. The bipedal edema improved, and her blood glucose remained well‐controlled despite the discontinuation of the DPP‐4i.

**FIGURE 1:**
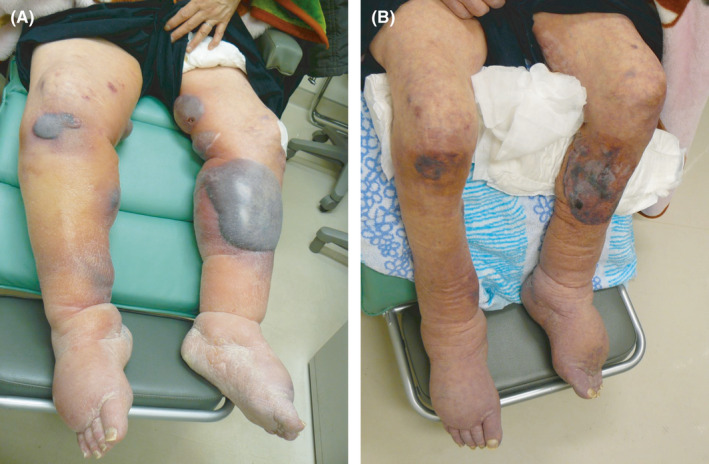
Photographs of the patient's lower extremities at (A) the time of the first visit to our hospital and (B) 1 week after discontinuing sitagliptin [Colour figure can be viewed at wileyonlinelibrary.com]

This case report includes two important messages. First, adverse drug events should be considered for skin rashes and edema of uncertain cause. Although some reports have been presented in relation to DPP‐4i‐associated bullous pemphigoid^1^ or edema,^2^ none present a case with severe blood blisters and edema. The mechanism of blister formation induced by DPP‐4i has not been sufficiently clarified. However, it may trigger some immunopathogenic reactions; in the case of DPP4i‐associated BP, an association with HLADQB1*03:01 has been noted.^3^ The possibility of adverse drug events affecting the skin has been reported with a variety of drugs.^4^ Therefore, primary care physicians should be aware of all medications in elderly patients, who can easily become polypharmatic, and should be cautious of unexpected, nonspecific adverse drug events, especially for new drugs with limited information on adverse effects. Second, physicians should seek to obtain information about when the patient began taking each drug to develop reasonable diagnostic hypotheses. Typically, older adults with decreased mental faculties are unable to provide accurate medical histories. Furthermore, in prolonged illnesses, the information recognized by doctors, family members, caregivers, and pharmacists can be fragmented. In Japan, patients usually have a pharmacy notebook^5^; however, sometimes old records are not preserved. Primary care physicians should focus on collaborating extensively with medical professionals and families to obtain patients' drug histories for more accurate diagnosis and treatment.

## CONFLICT OF INTEREST

The authors have stated explicitly that there are no conflicts of interest in connection with this article.

## AUTHOR CONTRIBUTIONS

HM conceived the idea and wrote the original draft of the manuscript. TK developed the theory, and HO and HK supervised the findings of this study. All authors discussed the case and commented on the manuscript. HM, TK, and TA revised and edited the manuscript. All authors have approved the final manuscript before submission.

## INFORMED CONSENT

We obtained written informed consent from the patient to publish this manuscript.
